# Utilizing Pharmacogenetic Results to Optimize Medication Management in Hospice Care: A Pilot Study

**DOI:** 10.3390/jpm15110543

**Published:** 2025-11-08

**Authors:** Erika N. Dreikorn, Carolyn Maxwell, Kayla Rowe, Brianna Brooks, Christine Munro, Natasha Robin Berman, Lucas A. Berenbrok, Mylynda B. Massart

**Affiliations:** 1 Clinical Translational Science Institute, University of Pittsburgh, Pittsburgh, PA 15213, USA; end20@pitt.edu; 2 Department of Human Genetics, School of Public Health, University of Pittsburgh, Pittsburgh, PA 15261, USA; cmaxwell@unmc.edu (C.M.); nkr10@pitt.edu (N.R.B.); 3 Department of Genetic Medicine, Munroe-Meyer Institute, University of Nebraska Medical Center, Omaha, NE 68106, USA; 4 Department of Pharmacy and Therapeutics, School of Pharmacy, University of Pittsburgh, Pittsburgh, PA 15261, USAberenbro@pitt.edu (L.A.B.); 5 School of Medicine, University of Pittsburgh, Pittsburgh, PA 15213, USA; 6 Department of Family Medicine, School of Medicine, University of Pittsburgh, Pittsburgh, PA 15213, USA

**Keywords:** pharmacogenomics (PGx), clinical pharmacogenetics, medication management, hospice care, palliative care, precision medicine, personalized medicine, gene–drug interactions, CPIC guidelines

## Abstract

**Background**: Pharmacogenetics (PGx), which examines how genetic variations influence drug metabolism and response, offers promise in hospice care where patients commonly experience polypharmacy, complex symptoms, and limited life expectancy. This study assessed the utility of PGx results in guiding medication adjustments to improve symptom management at the end of life. **Methods**: A retrospective chart review was conducted on ten patients enrolled in a Precision Hospice Program who had PGx results for six key metabolic genes. A PGx-trained pharmacist reviewed Clinical Pharmacogenetics Implementation Consortium (CPIC) guideline-based recommendations, which were discussed during interdisciplinary hospice team meetings. **Results**: Patients had a mean age of 85.7 years and were prescribed an average of 17.9 medications. Among the 27 prescriptions reviewed, actionable gene–drug interactions were identified, primarily involving antidepressants and analgesics. Three patients underwent medication changes based on PGx guidance, including switching from citalopram to bupropion and adding morphine to tramadol therapy, which improved symptom control. **Conclusion**: While not yet routinely implemented in hospice settings, this pilot study suggests PGx-guided prescribing can support personalized medication decisions and enhance emotional and physical comfort in end-of-life care when test results are available.

## 1. Introduction

Pharmacogenetics (PGx) examines how genetic variations in drug metabolism, transport, and target sites affect individual responses, aiming to improve medication safety and effectiveness [1]. These variations often occur in genes encoding drug-metabolizing enzymes, drug transporters, and drug targets, which can alter the speed at which drugs are activated, inactivated, or cleared. Depending on the patient’s genotype, these changes may result in reduced efficacy, increased risk of adverse drug events, or toxicity. By tailoring drug selection and dosing to a patient’s unique genetic profile, PGx can help clinicians reduce the likelihood of adverse drug reactions and improve therapeutic outcomes [2,3]. These benefits are particularly relevant in populations with complex medication regimens, where small improvements in tolerability and therapeutic match can have substantial impacts on quality of life.

PGx testing often focuses on polymorphisms in cytochrome p450 enzymes, which influence the metabolism of many commonly used hospice and palliative care medications. These enzymes determine how quickly a drug is cleared from the body or whether an inactive prodrug is effectively converted to its active form. For example, analgesics, such as codeine and tramadol, are activated by the CYP2D6 enzyme; CYP2D6 poor metabolizers ineffectively activate the drug and may experience reduced analgesia, while ultrarapid metabolizers have an increased risk of toxicity [4,5]. Conversely, antidepressants such as citalopram and escitalopram are inactivated by the CYP2C19 enzyme; rapid and ultrarapid metabolizers inactivate the drug too quickly and reduced response to the drug, while poor metabolizers have a greater toxicity risk [4,5]. Understanding these genotype–drug relationships is critical for tailoring medication regimens that align with the comfort-focused goals of hospice and palliative care, particularly given that many of the medications commonly used in these settings have published pharmacogenomic guidelines to support evidence-based prescribing.

In hospice and palliative care settings, the clinical goal shifts from curative treatment to maximizing comfort, symptom relief, and quality of life during the final stages of illness. Patients in these settings often contend with multiple comorbid conditions, communication barriers, and high rates of polypharmacy [6,7]. These factors increase the risk of medication-related complications at a time when minimizing harm is essential. Although tools such as PGx exist to support individualized prescribing, they remain underutilized in hospice care. One recent review of PGx in palliative settings found that testing is both feasible and acceptable, with up to half of patients having at least one actionable gene–drug interaction, though clinician adoption varied and economic evaluations were lacking [8]. By identifying gene–drug interactions that impact drug safety and efficacy, PGx provides an opportunity to optimize medication regimens in a way that aligns with patients’ goals of care and enhances comfort at the end of life.

The use of PGx in other specialties—such as oncology, psychiatry, and cardiology—has grown steadily in recent years, aided by expanding evidence-based guidelines from organizations like the Clinical Pharmacogenomics Implementation Consortium (CPIC) [4,5,9,10,11,12]. However, its role in hospice remains underexplored, and unique challenges to its implementation exist. Hospice patients may be physically frail, making sample collection difficult; test turnaround time must be short to make results meaningful; and recommendations must be balanced with patient and family preferences and overall care goals. Despite these challenges, preliminary studies in related settings suggest that PGx testing is both feasible and acceptable [5,13], and that it can meaningfully inform prescribing decisions. One feasibility study involving 100 hospice or palliative patients, in which a pharmacist-directed PGx decision support system led clinicians to adapt treatment regimens in more than half of cases, and most found the reports useful for improving care quality [14]. These findings highlight the promise of PGx in hospice care, but underscore the need for further research to understand its impact on medication optimization, symptom management, and deprescribing in patients nearing the end of life.

To help fill these gaps, this pilot study evaluated the real-world clinical utility of PGx in a hospice setting. We conducted a retrospective chart review to assess the impact of genotype-informed recommendations, guided by Clinical Pharmacogenetics Implementation Consortium (CPIC) guidelines, on prescribing decisions, medication burden, and symptom management for patients receiving end-of-life care.

## 2. Materials and Methods

This pilot project was conducted in collaboration with the Bethany Precision Hospice Program in southwestern Pennsylvania, which was launched in 2022 with the goal of enhancing end-of-life comfort care by utilizing PGx results for their hospice patients. PGx testing was performed using an expanded next-generation sequencing (NGS)–based pharmacogenomic panel that analyzes multiple pharmacogenes involved in drug metabolism and transport. The assay detects single-nucleotide variants (SNVs) and small insertions/deletions in clinically relevant pharmacogenes with high analytical sensitivity and specificity and was performed in a CLIA-certified, CAP-accredited laboratory. Although the full panel assesses a broader range of genes, only six—CYP2C19, CYP2C9, CYP2D6, CYP4F2, SLCO1B1, and VKORC1—were included in this analysis, as these genes had the most direct relevance to medications commonly used in hospice care. A hospice PGx-trained pharmacist and the medical director reviewed the clinical PGx results and made recommendations for dose or medication class changes, which were then reviewed and documented by the hospice team during weekly interdisciplinary group (IDG) meetings.

Due to limited program funding, PGx testing was available for only 11 hospice patients during the study period. One sample failed processing, leaving 10 patients for this retrospective chart review. Patients were considered eligible if they were estimated to live at least one month from the time of sample collection due to test turnaround time and to ensure test results could be used for medication management. In addition, patients needed to provide verbal consent for testing and be on at least one medication with a CPIC level A or B gene–drug guideline identified from their active medication list, as defined by the publicly available CPIC guidelines at the time of this study. Patient eligibility was screened during weekly IDG meetings, and individuals were enrolled on a rolling basis until 11 patients were identified.

Information collected from the chart review included patient demographics, medication histories, PGx results, and documented medication changes by the IDG hospice team. At the time of this study window, there were an additional 38 patients enrolled in the hospice program who did not undergo PGx testing. Among these 38 patients, the average number of medications with clinically actionable guidelines was 1.9, compared to 2.7 among the PGx-tested population included in this retrospective chart review. During the preparation of this work the authors used ChatGPT-4 to improve readability and language. After using this tool, the authors reviewed and edited the content as needed and take full responsibility for the content of the publication.

## 3. Results

In this study, patients had a mean age of 85.7 years, ranging from 75 to 92 years old. The cohort was representative of the population of western Pennsylvania, which is predominantly of European descent. Their hospice stay duration varied, with a mean length of stay (LOS) of 261.1 days, ranging from 56 to 515 days, and a median LOS of 201.5 days. Details on the primary indication for hospice admission and the number of comorbid diagnoses are provided in Table 1. On average, patients had 17.9 comorbid diagnoses (median = 14.5), with a range from 5 to 37. Patients were prescribed an average of 17.9 medications during their hospice stay, with a median of 18 medications. The number of prescribed drugs ranged from 11 to 29 (Table 1).

Among the ten patients included in this chart review, there were 15 unique medications over 27 total prescriptions with clinically actionable gene–drug interaction recommendations by CPIC (Table 2). Each patient had an average of 2.7 medications with clinically actionable gene–drug guidelines for consideration, with a median of 3 and ranging from 1 to 4 (Table 1). Table 2 provides details on the total number of patients taking each medication and the gene that affects its metabolism. The distribution of phenotypes among the 10 patients with PGx results can be found in Table A1. Interestingly, seven patients were classified as CYP2C19 rapid metabolizers, a proportion notably higher than the expected population frequencies of approximately 14% in American and 27% in European ancestry groups, as reported in the CPIC population allele frequency table. Given the small sample size, we did not conduct statistical analyses; however, this overrepresentation likely reflects sampling variation rather than a true difference in allele prevalence.

Table 3 summarizes the PGx-directed medication recommendations made by the hospice team. Most of these gene–drug interactions (19/27, 70%) did not result in medication changes. In 12 of these 19 cases, the current medications were compatible with the patients’ phenotypes and did not require adjustment. Additionally, 7 of these patients were already receiving therapeutic doses of their medications, so no changes were necessary in those instances. The PGx-guided medication evaluation led the hospice team to identify 5 prescriptions that were no longer beneficial to patients in hospice care, unrelated to PGx results. This included deprescribing statins in 4 cases and discontinuing duplicate therapy in 1 case.

Importantly, three out of ten patients with actionable PGx results underwent medication changes. This included patients 1 and 4, both CYP2C19 rapid metabolizers, who were de-prescribed citalopram or escitalopram based on CPIC recommendations and transitioned to bupropion therapy. In addition, patient 5, a CYP2D6 poor metabolizer, continued tramadol due to therapeutic relief but morphine (a non-codeine opioid) was added for supplemental as-needed pain control. According to nurse case-manager documentation, patients who underwent medication adjustments reported improved symptom control and overall comfort; however, these outcomes were reported qualitatively and were not formally assessed as part of this pilot study.

## 4. Discussion

This pilot study aimed to address the gap in understanding the clinical utility of pharmacogenetics (PGx) in hospice care and its potential to optimize medication management and improve patient care at the end of life. Findings from this chart review suggest that PGx had its greatest impact on medications related to comfort, particularly in pain control and emotional well-being, which aligns with the goals of hospice care. These preliminary data indicate that PGx results, when available to inform guided prescribing decisions, can be a valuable tool in hospice care, thereby enhancing patient outcomes and quality of life.

The medication classes prescribed to the patients in this study were consistent with previously published essential palliative care medications [6]. PGx results identified numerous gene–drug interactions, with actionable prescribing recommendations for several patients, primarily related to antidepressants and analgesics, addressing key aspects of both physical and emotional comfort in hospice. Beyond identifying drug–gene interactions that prompt medication changes, PGx testing in hospice care may also support deprescribing decisions. In this study, several medications were discontinued because they were no longer aligned with the patient’s goals of care, even when not directly related to PGx results.

Overall, three out of ten patients with actionable PGx results underwent medication changes. Two patients were switched to bupropion due to poor responses to citalopram and escitalopram, and one patient was prescribed morphine in addition to tramadol based on PGx recommendations that this patient, A CYP2D6 poor metabolizer, would be expected to ineffectively convert tramadol (a prodrug) to its active metabolite. According to hospice nurse documentation, these patients experienced subjective improvement in symptom control and overall comfort following their medication changes; however, symptom improvement was not qualitatively assessed in this study. Together, these findings highlight the potential of PGx to personalize hospice care and enhance patient comfort and well-being.

Some considerations and barriers to PGx testing in hospice care include challenges in obtaining samples, as saliva is not easily collected from older and ill patients, and blood draws are considered invasive. Additionally, the timing of PGx testing emerged as an important consideration for maximizing clinical utility. In settings where results may take several days or weeks to return, actionable findings may arrive too late to influence symptom management decisions. To address these timing challenges, patients would likely benefit from preemptive PGx testing before hospice admission. Integrating PGx testing early in the hospice admission process, or preemptively in patients at high risk of transitioning to hospice, could ensure results are available when needed most. Such an approach may enable providers to make immediate, genotype-informed medication adjustments and avoid prolonged periods of suboptimal symptom control.

The implementation of pharmacogenomics in older adult populations remains limited, despite the high prevalence of polypharmacy, multimorbidity, and age-related changes in drug metabolism. These factors increase the risk of adverse drug events and highlight the potential value of incorporating PGx-guided prescribing into hospice and geriatric care. However, barriers such as workflow integration, cost, informatics infrastructure, and the need for clinician education continue to hinder adoption in geriatric medicine [15]. Our findings reflect similar challenges within the hospice setting, where the benefits of PGx may be substantial but practical implementation remains constrained by system-level and educational factors. Addressing these barriers will be critical to expanding access to PGx-guided care among older adults and improving medication safety and quality of life at the end of life.

Successful integration of PGx into hospice care also depends on the knowledge and comfort level of the interdisciplinary team members interpreting and applying the results. While pharmacists are well positioned to analyze complex drug–gene relationships, ensuring consistent understanding among physicians, nurses, and other team members is critical. Expanding PGx education across hospice staff could enhance uptake and support more confident clinical decision-making. In addition, incorporating PGx decision-support tools into electronic health records may reduce the cognitive burden on clinicians and provide accessible, evidence-based recommendations at the point of care. For example, an implementation program in pain management and primary care integrated pharmacist expertise with EHR-based decision support, successfully guiding the use of opioids and antidepressants—two drug classes frequently prescribed in hospice care. Adapting similar strategies for end-of-life settings could improve workflow efficiency and promote consistent, evidence-based adoption of PGx in hospice practice [16]. In addition, future research could benefit from integrating expanded PGx panels to capture a wider range of gene–drug interactions as well as using multiple pharmacogenomic knowledgebases—such as PharmGKB and PharmVar—alongside CPIC guidelines, to broaden variant interpretation and enhance the robustness of gene–drug insights applied in hospice care.

A key limitation of this study is the small sample size of only ten patients, which restricts the generalizability of the findings and underscores the need for larger-scale investigations. Future research should focus on evaluating PGx-guided prescribing in larger, more diverse hospice populations and across multiple care settings. Studies combining quantitative outcomes with qualitative insights from patients, caregivers, and providers could clarify the full impact of PGx on symptom control, adverse event reduction, and care satisfaction for both the patient and provider. Importantly, the design of such studies should prioritize minimal disruption to existing clinical workflows—as exemplified by the “Supportive Care PGx Trial” in oncology, which successfully embedded PGx testing and pharmacist review into routine symptom management without altering clinic operations [4].

Cost–effectiveness analyses are also warranted to assess whether reductions in medication-related complications offset testing costs, to inform the scalability of PGx implementation in hospice care. Notably, a recent review of PGx in palliative settings found that, although several studies demonstrated the feasibility and acceptability, none included an economic evaluation—highlighting a critical gap in the literature [8].

## 5. Conclusions

This study offers promising early evidence that PGx can enhance hospice care by supporting personalized medication management that promotes emotional and physical comfort. Although limited by a small sample size and retrospective design, the findings demonstrate the feasibility and potential clinical value of integrating PGx into routine hospice workflows. Expanding access to PGx testing, improving clinician education, and embedding decision-support tools in clinical systems represent key next steps toward implementing precision medicine approaches that improve the safety, comfort, and quality of end-of-life care.

## Figures and Tables

**Table 1:** Patient demographics.

Patient	Primary Indication for Hospice Admission	Co-Morbid Diagnoses (N )	Age	LOS ^a^	# Meds. Prescribed	# Meds. with Clinically Actionable Guidelines
1	Atherosclerotic Heart Disease of Native Coronary Artery without Angina Pectoris	37	86	355	19	2
2	Alzheimer’s Disease, Unspecified	13	80	222	22	4
3	Chronic Kidney Disease, Stage 5	12	75	154	18	1
4	Chronic Respiratory Failure with Hypoxia	27	92	181	18	4
5	Chronic Respiratory Failure with Hypoxia	29	90	384	14	3
6	Chronic Respiratory Failure with Hypoxia	12	82	125	19	2
7	Alzheimer’s Disease, Unspecified	5	92	454	13	3
8	Chronic Diastolic (Congestive) Heart Failure	20	89	165	29	3
9	Alzheimer’s Disease, Unspecified	8	89	515	16	2
10	Hemiplegia Following Cerebral Infarction Left Dominant Side	16	82	56	11	3
mean		17.9	85.7	261.1	17.9	2.7
median		14.5	87.5	201.5	18	3
range		5–37	75–92	56–515	11–29	1–4

^a^ LOS = Length of stay. # Meds. = number of medications.

**Table 2:** Pharmacogenomic implications for medication use in hospice patients.

Drug Classification (Total)	Drug	Patients Prescribed	Associated Gene on PGx Panel	CPIC Level of Evidence
Antidepressant (8)	Citalopram	1	*CYP2C19*	A
	Escitalopram	3	*CYP2C19*	A
	Sertraline	1	*CYP2C19*	A
	Paroxetine	2	*CYP2D6*	A
	Venlafaxine	1	*CYP2D6*	B
Analgesic (6)	Ibuprofen	1	*CYP2C9*	A
	Meloxicam	1	*CYP2C9*	A
	Tramadol	3	*CYP2D6*	A
	Hydrocodone	1	*CYP2D6*	B
Cardiovascular Agents (6)	Atorvastatin	3	*SLCO1B1*	A
	Pravastatin	2	*SLCO1B1*	A
	Simvastatin	1	*SLCO1B1*	A
Gastrointestinal Agents (4)	Omeprazole	1	*CYP2C19*	A
	Pantoprazole	3	*CYP2C19*	A
Antiemetics (3)	Ondansetron	3	*CYP2D6*	A

**Table 3:** CPIC therapeutic recommendations and medication changes based on gene–drug interactions identified by PGx testing.

Patient #	Gene and Genotype (AS) ^a^	Phenotype	Drug	CPIC ^b^ Therapeutic Recommendations	Recommendation Classification	PGx-Directed Medication Change
1	CYP2C19 *1/*17	Rapid metabolizer	Citalopram	Initiate therapy with recommended starting dose. If patient does not adequately respond to recommended maintenance dosing, consider titrating to a higher maintenance dose or switching to a clinically appropriate alternative antidepressant not predominantly metabolized by CYP2C19.	Optional	Changed to Bupropion ^e^
	CYP2D6 *1/*1 (2.0)	Normal metabolizer	Tramadol	Use tramadol label recommended age specific or weight-specific dosing.	Strong	No change Indicated ^c^
2	CYP2C19 *1/*2	Intermediate metabolizer	Omeprazole	Initiate standard starting daily dose. For chronic therapy (>12 weeks) and efficacy achieved, consider 50% reduction in daily dose and monitor for continued efficacy.	Optional	Patient therapeutic, no change Indicated ^c^
			Pantoprazole	Initiate standard starting daily dose. For chronic therapy (>12 weeks) and efficacy achieved, consider 50% reduction in daily dose and monitor for continued efficacy.	Optional	Identified duplicate therapy, discontinued pantoprazole ^d^
	CYP2D6 *1/*1 (2.0)	Normal metabolizer	Tramadol	Use tramadol label recommended age specific or weight-specific dosing.	Strong	No change Indicated ^c^
	*SLCO1B1 rs4149056 TT*	Normal function	Pravastatin	Prescribe desired starting dose and adjust doses based on disease-specific guidelines from 2022 CPIC guideline.	Strong	No change Indicated ^c^
3	CYP2D6 *4/*41 (0.25)	Intermediate metabolizer	Hydrocodone	Use hydrocodone label recommended age-specific or weight-specific dosing. If no response and opioid use is warranted, consider non-codeine or non-tramadol opioid.	Optional	Patient therapeutic, no change Indicated ^c^
4	CYP2C19 *1/*17	Rapid metabolizer	Escitalopram	Initiate therapy with recommended starting dose. If patient does not adequately respond to recommended maintenance dosing, consider titrating to a higher maintenance dose or switching to a clinically appropriate alternative antidepressant not predominantly metabolized by CYP2C19.	Optional	Change to Bupropion ^e^
	CYP2C9 *1/*1 (2.0)	Normal metabolizer	Meloxicam	Initiate therapy with recommended starting dose. In accordance with the prescribing information, use the lowest effective dosage for shortest duration consistent with individual patient treatment goals.	Strong	No change Indicated ^c^
	CYP2D6 *1/*1 (2.0)	Normal metabolizer	Ondansetron	Initiate therapy with recommended starting dose.	Strong	No change Indicated ^c^
	*SLCO1B1 rs4149056 TT*	Normal function	Atorvastatin	Prescribe desired starting dose and adjust doses based on disease-specific guidelines from 2022 CPIC guideline.	Strong	No change Indicated ^c^
5	CYP2C9 *1/*3 (1.0)	Intermediate metabolizer	Ibuprofen	Initiate therapy with lowest recommended starting dose. Titrate dose upward to clinical effect or maximum recommended dose with caution. In accordance with the prescribing information, use the lowest effective dosage for shortest duration consistent with individual patient treatment goals. Carefully monitor adverse events, such as blood pressure and kidney function during course of therapy.	Moderate	No change Indicated ^c^
	CYP2D6 *4/*4 (0)	Poor metabolizer	Paroxetine	Consider a 50% reduction in recommended starting dose, slower titration schedule, and a 50% lower maintenance dose as compared with normal metabolizers.	Moderate	Patient therapeutic, no change Indicated ^c^
			Tramadol	Avoid tramadol use because of possibility of diminished analgesia. If opioid use is warranted, consider a non-codeine opioid.	Strong	Prescribed morphine in addition to tramadol ^e^
6	CYP2C19 *1/*1	Normal metabolizer	Escitalopram	Initiate therapy with recommended starting dose.	Strong	No change Indicated ^c^
	*SLCO1B1 rs4149056 CT*	Decreased function	Pravastatin	Prescribe desired starting dose and adjust doses of pravastatin based on disease-specific guidelines. Prescriber should be aware of possible increased risk for myopathy with pravastatin especially with doses > 40 mg per day.	Moderate	Discontinued, as no further benefit on hospice ^d^
7	CYP2D6 *3/*41 (0.25)	Intermediate metabolizer	Paroxetine	Consider a lower starting dose and slower titration schedule as compared with normal metabolizers.	Optional	Patient therapeutic, no change Indicated ^c^
			Ondansetron	Insufficient evidence demonstrating clinical impact based on CYP2D6 genotype. Initiate therapy with recommended starting dose.	No recommendation	No change Indicated ^c^
	*SLCO1B1 rs4149056 TT*	Normal function	Atorvastatin	Prescribe desired starting dose and adjust doses based on disease-specific guidelines from 2022 CPIC guideline.	Strong	Discontinued, as no further benefit on hospice ^d^
8	CYP2C19 *1/*17	Rapid metabolizer	Pantoprazole	Initiate standard starting daily dose. Consider increasing dose by 50–100% for the treatment of Helicobacter pylori infection and erosive esophagitis. Daily dose may be given in divided doses. Monitor for efficacy.	Moderate	Patient therapeutic, no change Indicated ^c^
	CYP2D6 *1/*1 (2.0)	Normal metabolizer	Venlafaxine	Initiate therapy with recommended starting dose.	Strong	No change Indicated ^c^
			Ondansetron	Initiate therapy with recommended starting dose.	Strong	No change Indicated ^c^
9	CYP2C19 *1/*17	Rapid metabolizer	Sertraline	Initiate therapy with recommended starting dose.	Strong	No change Indicated ^c^
	*SLCO1B1 rs4149056 TT*	Normal function	Atorvastatin	Initiate therapy with recommended starting dose.	Strong	Discontinued, as no further benefit on hospice ^d^
10	CYP2C19 *1/*17	Rapid metabolizer	Escitalopram	Initiate therapy with recommended starting dose. If patient does not adequately respond to recommended maintenance dosing, consider titrating to a higher maintenance dose or switching to a clinically appropriate alternative antidepressant not predominantly metabolized by CYP2C19.	Optional	Patient therapeutic, no change Indicated ^c^
			Pantoprazole	Initiate standard starting daily dose. Consider increasing dose by 50–100% for the treatment of Helicobacter pylori infection and erosive esophagitis. Daily dose may be given in divided doses. Monitor for efficacy.	Moderate	Patient therapeutic, no change Indicated ^c^
	*SLCO1B1 rs4149056 TT*	Normal function	Simvastatin	Prescribe desired starting dose and adjust doses based on disease-specific guidelines from 2022 CPIC guideline.	Strong	Discontinued, as no further benefit on hospice ^d^

^a^ AS = activity score, when applicable. ^b^ CPIC = Clinical Pharmacogenetics Implementation Consortium. ^c^ No change to medication. ^d^ Medication discontinued. ^e^ Medication change.

## Data Availability

The original contributions presented in this study are included in the article. Further inquiries can be directed to the corresponding author.

## Abbreviations

The following abbreviations are used in this manuscript:

PGx	Pharmacogenomics/Pharmacogenetics
CPIC	Clinical Pharmacogenetics Implementation Consortium
IDG	Interdisciplinary Group
IRB	Institutional Review Board
LOS	Length of Stay
AS	Activity Score

## Appendix A

**Table A1:** Distribution of metabolizer and function status for pharmacokinetic and pharmacodynamic genes associated with hospice drugs among patients.

Metabolizer Status	Ultrarapid	Rapid	Normal	Intermediate	Poor
CYP2C19		7	2	1	
CYP2C9			5	5	
CYP2D6	1		4	3	2
CYP4F2			6	4	
**Function Status**	**Increased**	**Normal**		**Decreased**	
SLCO1B1		8		2	
VKORC1		7		3	
